# Risk Factors for Senile Corneal Arcus in Patients with Acute Myocardial Infarction

**Published:** 2010-10

**Authors:** Mirnaghi Moosavi, Ahmad Sareshtedar, Siamak Zarei-Ghanavati, Mehran Zarei-Ghanavati, Nazanin Ramezanfar

**Affiliations:** 1 Eye Research Center, Mashhad University of Medical Sciences, Khatam-al-Anbia Eye Hospital, Mashhad, Iran; 2 Department of Cardiology, Ghaem Hospital, Mashhad University of Medical Sciences, Mashhad, Iran

**Keywords:** Corneal Arcus, Hypercholesterolemia, Hyperlipidemia, Hypertriglyceridemia, Myocardial Infarction

## Abstract

**Purpose:**

To investigate the association between senile corneal arcus and atherosclerosis risk factors in patients with recent acute myocardial infarction.

**Methods:**

In this cross sectional study, atherosclerosis risk factors including fasting blood sugar, total cholesterol and triglyceride levels were measured in 165 patients with recent (less than three months’ duration) acute myocardial infarction. Slitlamp examination was performed to detect corneal arcus. Associations between senile corneal arcus and atherosclerosis risk factors were assessed.

**Results:**

Overall, 165 patients including 100 male and 65 female subjects with mean age of 62±10.3 years were evaluated. In 122 patients (74%), variable degrees of corneal arcus were observed. The presence of corneal arcus was significantly associated with age (P = 0.03) and high levels of total cholesterol (over 200 mg/dl, P < 0.01). After adjusting for age, arcus was not associated with sex (P = 0.10), hypertriglyceridemia (P = 0.09), fasting blood sugar (P = 0.06), or systemic hypertension (P = 0.08).

**Conclusion:**

Our study revealed that corneal arcus is associated with age and hypercholesterolemia in patients with recent acute myocardial infarction. No association was detected with sex, fasting blood sugar, hypertension, and hypertriglyceridemia.

## INTRODUCTION

Corneal arcus is a common feature of aging in the human eye and is due to deposition of lipids in the peripheral corneal stroma. The arcus has a hazy gray-white appearance, a sharp outer margin but an indistinct inner border.^1^ A lucid interval of Vogt is usually present between the peripheral edge of the arcus and the limbus.^2^,^3^ Senile arcus initially appears in the inferior and superior poles of the cornea, but involves the entire circumference of the cornea in later stages.^1^

Suggestions that corneal arcus is analogous to atherosclerosis were first made more than two centuries ago^2^; this seems logical due to histological similarities. The cornea and blood vessels are both composed of connective tissue in the middle zone, namely the corneal stroma and the media of the artery, containing a single cell type: fibroblasts and smooth muscle cells respectively. Lipid deposition in the cornea is visible and more readily studied, which adds extra interest to what is happening in the cornea.

Corneal arcus may occur without any predisposing factors, but several studies have reported associations between corneal arcus and cardiovascular risk factors.^4^,^5^ Reports have also linked corneal arcus with alcoholism^4^, diabetes mellitus^4^, high blood pressure^4^, smoking^6^, and obesity^4^. However, these associations remain controversial.

In this study, we evaluated the association between corneal arcus and hyperlipidemia in patients with recent acute myocardial infarction (MI) and explored possible associations with fasting blood sugar and hypertension.

## METHODS

Patients referred to Ghaem Hospital with recent (less than 3 months’ duration) acute MI, aged 30 years or more were evaluated. Informed consent was obtained from all study participants. The patients were evaluated using a standardized interviewer-administered questionnaire and underwent a standard eye examination. Fasting blood samples were collected for measurement of fasting blood sugar, total cholesterol and triglyceride levels.

Sex, age and medical history were recorded during the interview. Arcus was assessed by an ophthalmologist through slitlamp examination and categorized as absent, partial (involving less than 180 degrees of the cornea), or circumferential (involving 180 degrees or more). If corneal arcus was asymmetric in the two eyes, the higher grade was considered. Hypertension was defined as the concurrent use of antihypertensive medications, systolic blood pressure above 140 mmHg, or diastolic blood pressure above 90 mmHg at the time of examination. Diabetes was defined by a self-reported history of diabetes or fasting blood glucose levels higher than 110 mg/dl. Serum lipids were compared with the following reference levels: total cholesterol 200 mg/dl and triglyceride 160 mg/dl.

Statistical analysis was performed with the chi-square test utilizing SPSS software (SPSS Inc., Chicago, USA). P-values less than 0.05 were considered significant.

## RESULTS

One hundred sixty-five patients including 100 male and 65 female subjects with recent acute MI were evaluated. Mean age was 62±10.3 years. In 122 (74%) of the patients, variable degrees of corneal arcus were present. In all affected patients, the arcus was bilateral and more than 90% of subjects (112 patients) showed similar degrees of involvement.

Corneal arcus was not associated with sex (P=0.10). Mean age in patients with and without corneal arcus was 63±8.1 and 38±7.1 years, respectively. The presence of corneal arcus was positively correlated with age (P=0.03).

Corneal arcus was significantly associated with total cholesterol levels over 200 mg/dl (P<0.01). Mean total cholesterol in patients with and without corneal arcus was 250 and 190 mg/dl, respectively (P=0.02).

Mean fasting blood sugar levels in patients with and without corneal arcus was 104 versus 98 mg/dl (P=0.06), but after adjustment for age, arcus was not associated with high blood sugar. In addition, corneal arcus was not associated with hypertriglyceridemia (P=0.09). An association was observed between corneal arcus and hypertension but after adjusting for age, this association was not significant (P=0.08).

## DISCUSSION

To the best of our knowledge the current study is the first to evaluate corneal arcus in patients with recent acute MI. The appearance of arcus is characteristic and easily detected; therefore, our results can determine the significance of corneal arcus in patients at risk of severe coronary heart disease.

Early observers attributed corneal senile arcus to dryness of the cornea^7^, local vascular impairment^1^, or chronic inflammation^2^. Lawrence^8^ and Middlemore^9^ were among the first to associate arcus with atherosclerosis. In 1850, Canton^10^ demonstrated that the corneal deposits in arcus were insoluble in water and acetic acid but soluble in ether, suggesting it was lipid. Detailed histological observations by Cogan and Kuwabara^11^ demonstrated extracellular but limited intracellular lipids and absence of underlying degenerative changes, leading them to reject the hypothesis that arcus is a simple senile degeneration and to postulate that the lipids are derived from the blood.

The correlation between corneal arcus and serum lipid levels is supported by the lipid nature of the corneal deposit, an increased incidence of arcus in familial hyperlipidemia and a variety of association studies.^12^–^15^ Unesterified cholesterol deposits have been detected in the limbal cornea and conjunctiva of hypercholesterolemic rabbits using fluorescent dye.^16^ Regression of corneal arcus has been demonstrated in hyperlipidemic animal models following cessation of diet-induced hyperlipidemia,^17^ but such regression has not been demonstrated with lipid-lowering therapy in human subjects.

Rosenman et al^18^ in a retrospective analysis of the Western Collaborative Study reported that the incidence of myocardial infarction was increased in subjects with corneal arcus, but only in young men aged 39–49. The odds for coronary heart disease (CHD) in subjects 39–49 years of age with and without corneal arcus was 1.57 after adjusting for age, serum cholesterol, and smoking. These results suggest that corneal arcus may be an independent risk factor for CHD.

Klein et al^19^ used data from 2,530 Caucasians and observed a higher 7-year incidence of CHD in white men with corneal arcus than men without arcus. However, after adjusting for age, corneal arcus was no longer predictive of CHD.

Halfon et al^20^ prospectively followed 947 men and 972 women over 40 years of age for 20 years; CHD mortality was higher in subjects with baseline corneal arcus, but only in white men aged 40–59 (P<0.05).

In 1990, Chambless et al^21^, using the Lipid Research Clinics Mortality Follow-up Study data concluded that corneal arcus was a predictor of CHD, independent of total cholesterol, serum HDL cholesterol, and smoking among hyperlipidemic men aged 30–49 years (estimated relative risk of 6.7).

The results of the Framingham Heart Study also showed a direct association between corneal arcus and CHD.^22^

According to our results, a significant correlation exists between corneal arcus and age. Corneal arcus was not correlated with gender, although other studies have shown higher rates in men. Moreover, after adjusting for age, arcus senilis was not associated with blood pressure and fasting blood sugar levels. As expected, the association between hyperlipidemia and arcus was significant. Due to lack of a control group we could not evaluate the correlation between severe cardiovascular disease (presenting with acute MI) and corneal arcus. Further studies with a control group are required to elucidate this association.
